# Traumatic Intra-articular Intercondylar Dislocation of the Patella Reduced by the Closed Method with Open Quadriceps Repair in an Adolescent: A Rare Case Report

**DOI:** 10.7759/cureus.3705

**Published:** 2018-12-08

**Authors:** S K Venkatesh Gupta, Powdhan H P, R Prathik, Lamba Jeetesh

**Affiliations:** 1 Department of Orthopaedics, Jagadguru Jayadeva Murugarajendra Medical College, Davangere, IND

**Keywords:** patella, dislocation, quadriceps tendon, knee joint

## Abstract

Acute patellar dislocation is a common condition and is seen in situations that are associated with dance and sports. The intra-articular dislocation of the patella is a rare condition while lateral dislocation is most common. Two types of intra-articular dislocations are described in the literature, horizontal and vertical. The absolute management of acute patella dislocation following trauma in the young population is still in debate. Here, we present a 14-year-old male patient with traumatic intra-articular intercondylar dislocation of the patella reduced by the closed method with open quadriceps repair on the basis of ultrasound and magnetic resonance imaging (MRI).

## Introduction

Traumatic patella dislocation is an emergency condition. An average incidence of 29 per 1,00,000 population is reported most commonly among adolescents in the 10-17 year age group. Patella dislocation is mostly caused by physical activities, such as sports and dance [1]. Lateral dislocations of the patella are usually reduced spontaneously or by closed manipulation, just by extending the knee joint [2]. Intra-articular dislocation of the patella is a rare condition while the lateral type is the most common, as described in the literature. In very few incidences, the trauma causes the patella to rotate on its horizontal axis while the upper pole wedges into the intercondylar notch, resulting in intra-articular dislocation [3]. We present here a case of traumatic intra-articular intercondylar dislocation of the patella.

## Case presentation

**Figure 1:**
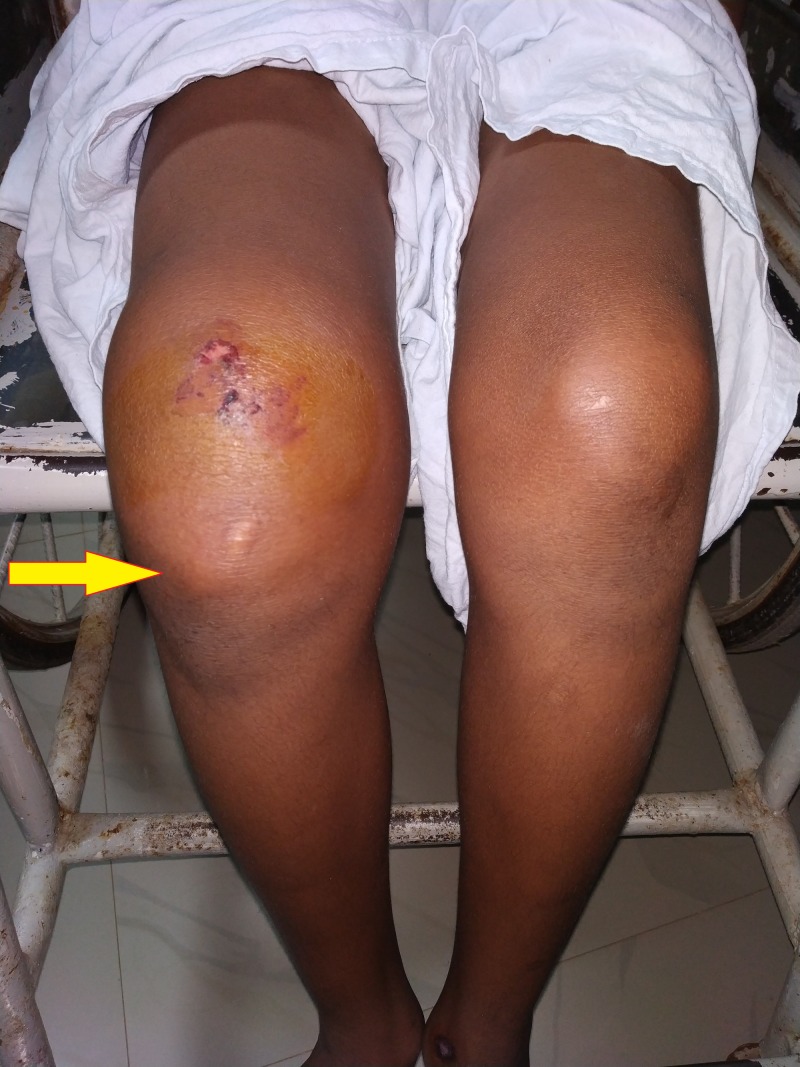
Presentation of the patient to casualty, swelling present in the right knee joint with prominent inferior pole of patella: anterior view

**Figure 2:**
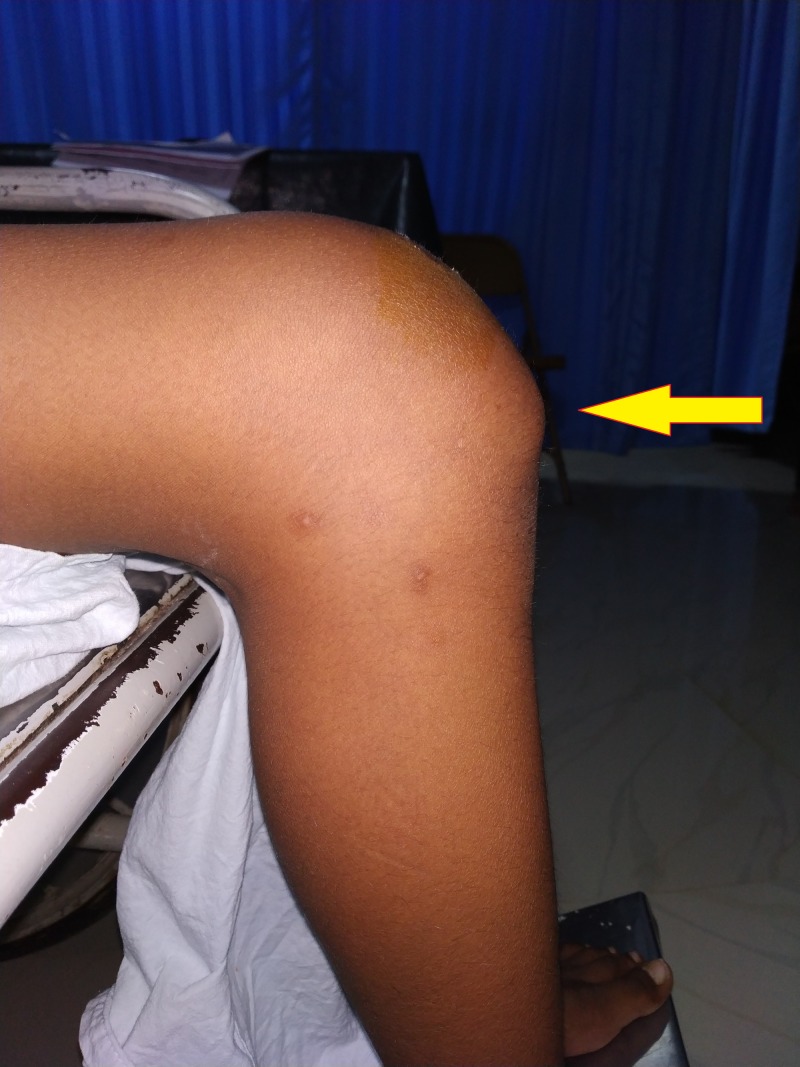
Inferior pole of the patella being more prominent: lateral view of the right knee

A 14-year-old adolescent male was brought to Chigateri Government Hospital Davangere, Karnataka, with an alleged history of self-falling on to a rock from a height of seven feet while playing and sustaining an injury to his right knee. Pain was sudden in onset, localized to the right knee, excruciating in nature, aggravated on movement, and relieved on immobilization. The patient was unable to walk. On local examination, swelling and an abrasion, measuring 1 cm in length, 2 cms in breadth, were present on the anterior aspect of the right knee. The skin over the swelling was stretched and shiny. The superior pole of the right patella was not palpable while the inferior pole was most prominent. The patellar tendon was taut and intact. The patient was comfortable in 85 degrees of flexion in the right knee (Figures 1-2). A further five to 10 degrees passive flexion was possible but was painful. Further tests were not performed due to the discomfort of the patient. The normal range of movements was present at the ipsilateral hip and ankle joint. There were no distal neurovascular deficits.

**Figure 3:**
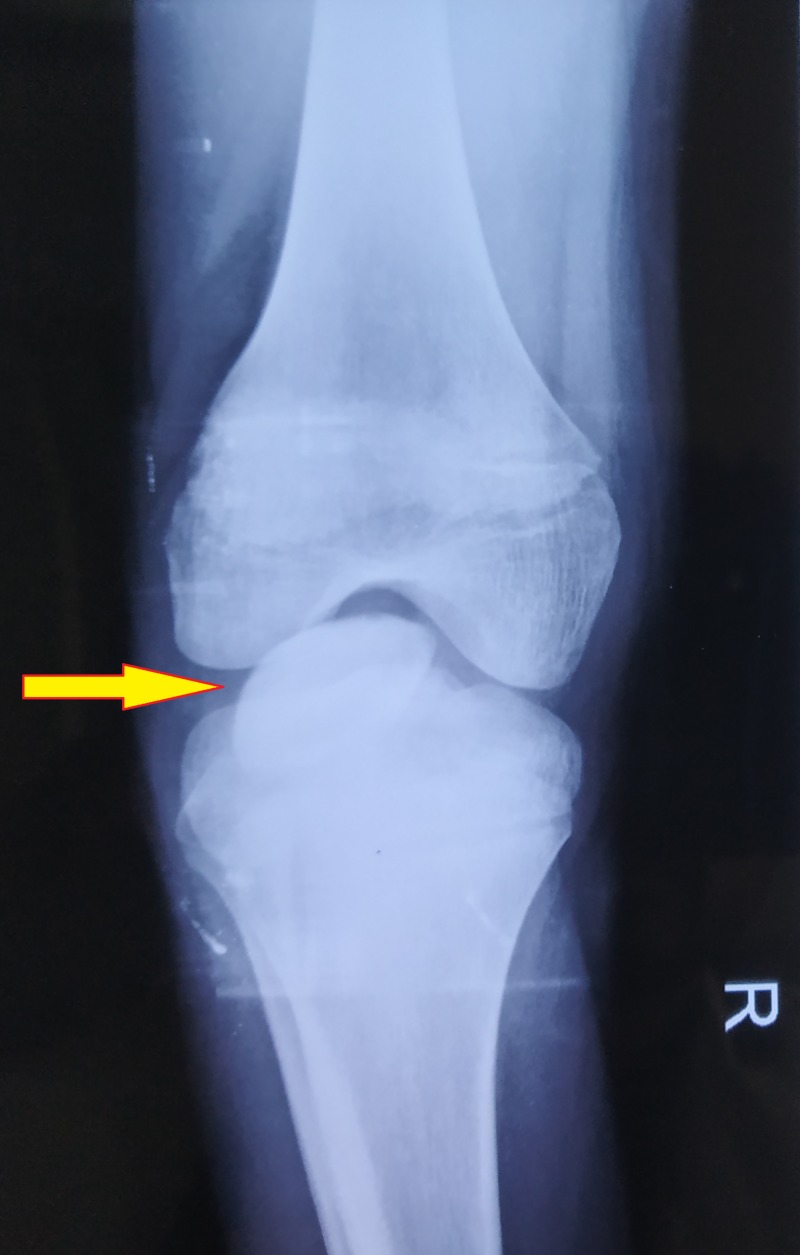
X-ray of the right knee shows intra-articular dislocation of the patella: anteroposterior view

**Figure 4:**
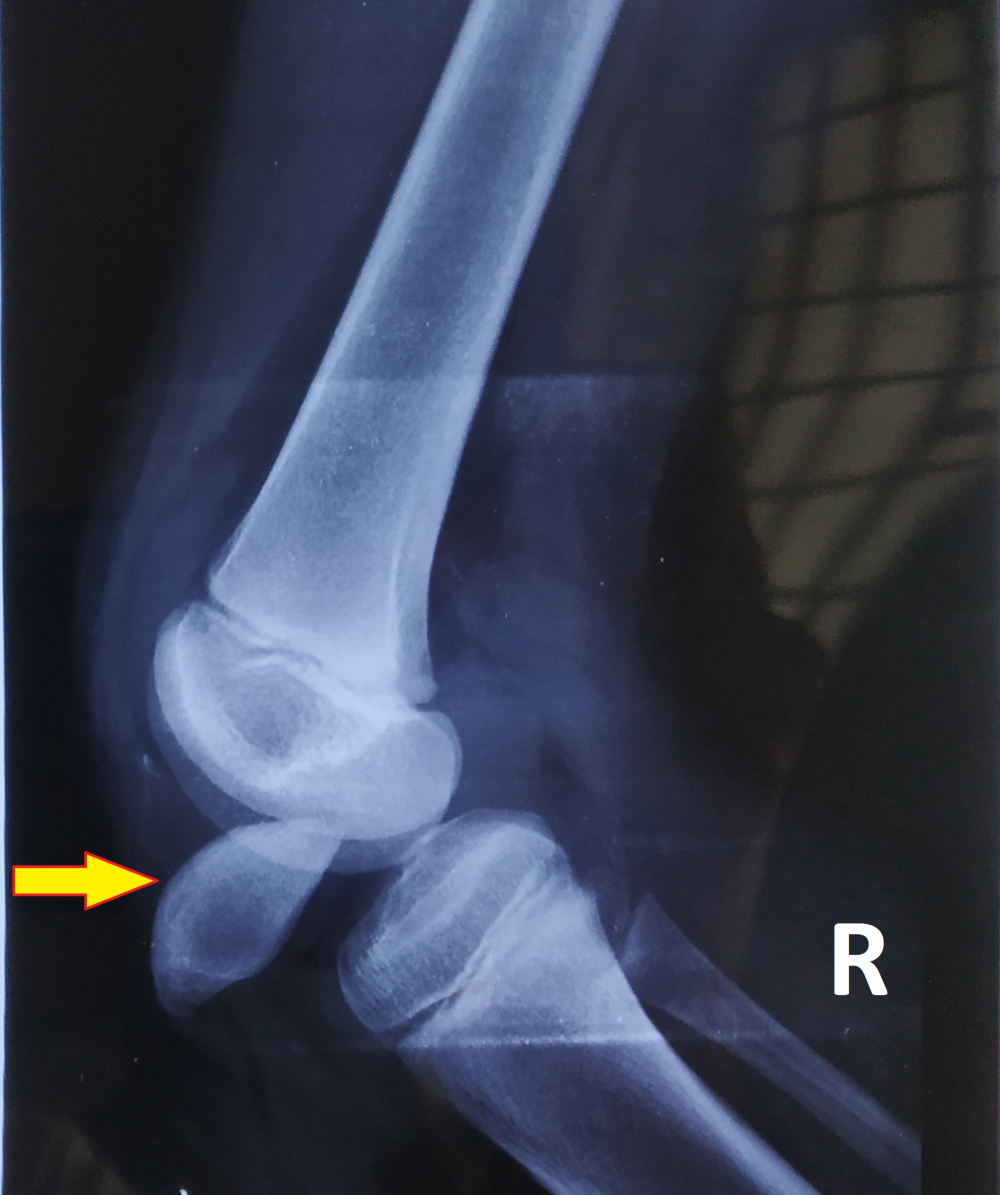
X-ray of the right knee shows intra-articular dislocation of the patella: lateral view

**Figure 5:**
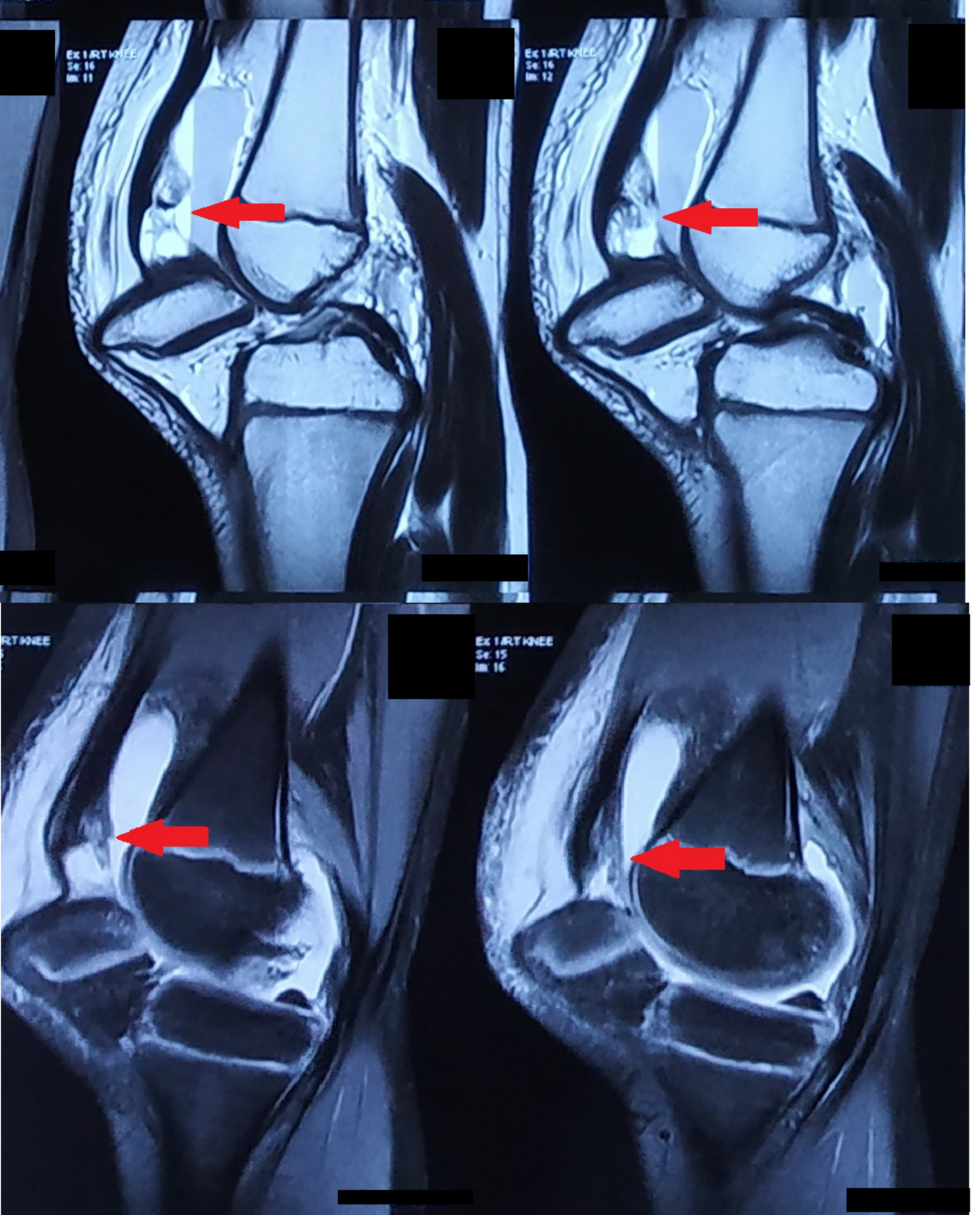
MRI scans of the right knee show a partial tear of the quadriceps tendon MRI: magnetic resonance imaging

Investigations

a. Blood investigations: Within normal limits.

b. X-ray of right knee: anteroposterior and lateral views (Figures 3-4) show intra-articular, intercondylar dislocation of the patella.

c. Emergency ultrasound report: Partial tear of the quadriceps tendon with minimal joint effusion.

d. MRI of the right knee joint was done (Figure 5), which reported intercondylar dislocation of the right patella oriented in the horizontal axis, buckling of the quadriceps tendon with a partial tear near the attachment of the patella, intra-substance edema in the anterior cruciate ligament, and supra-patellar bursa effusion with fluid level, denoting hemarthrosis.

The patient was immobilized with an above-knee slab until he was taken to the operation theater for further management.

Course of patella dislocation reduction

**Figure 6:**
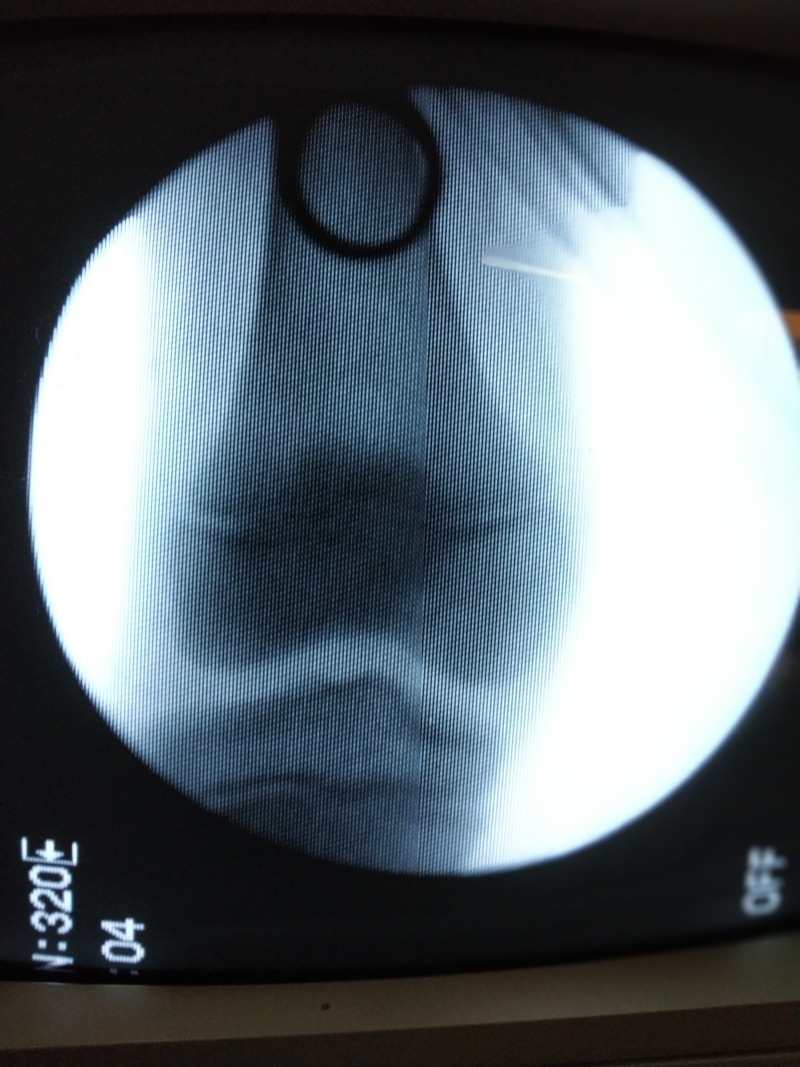
On-table immediate post-reduction of the patella visualized on the image intensifier: anteroposterior view

**Figure 7:**
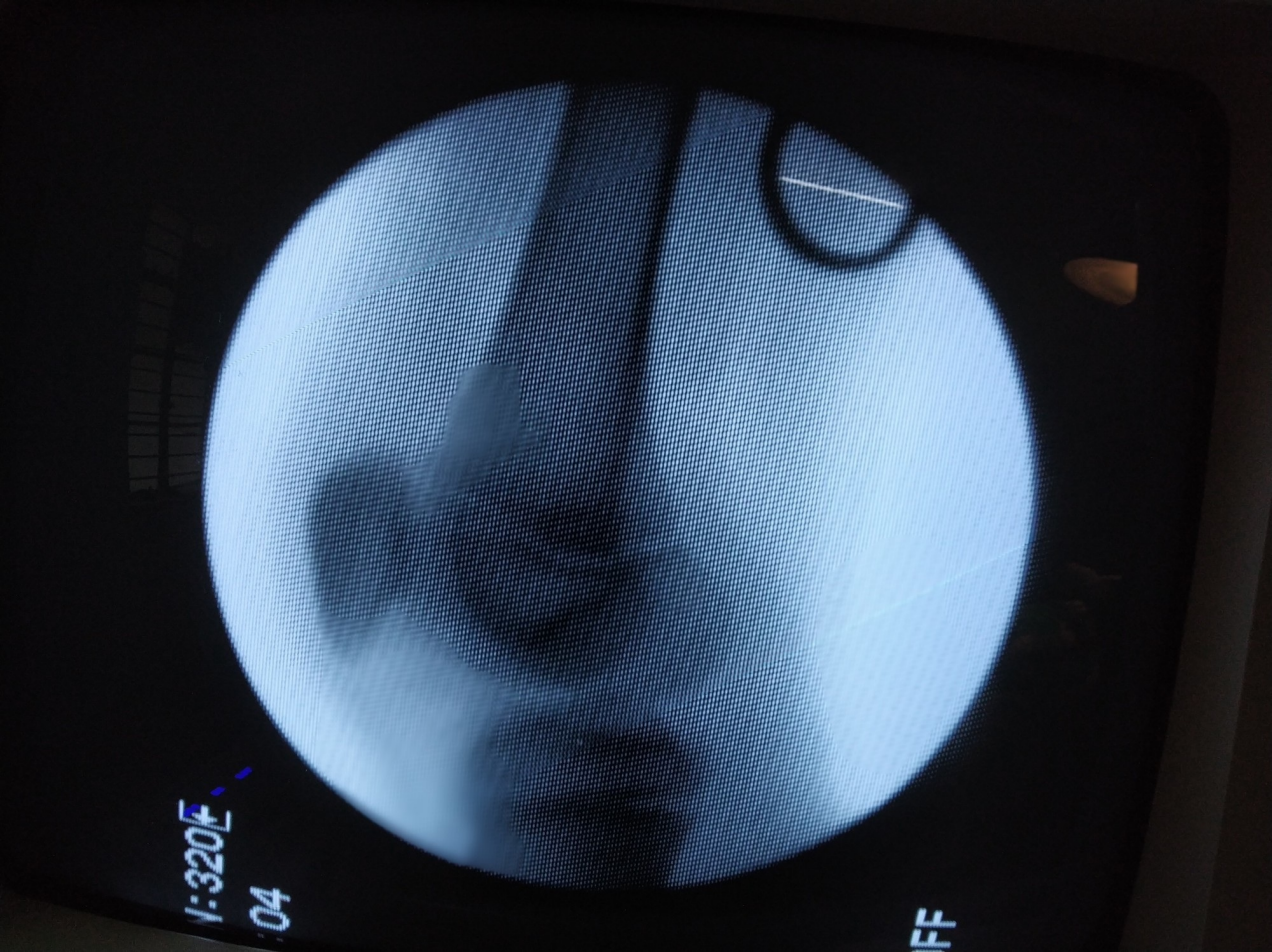
On-table immediate post-reduction of the patella visualized on the image intensifier: lateral view

Under general anesthesia with muscle relaxation, closed reduction was attempted and was successful at the first attempt. The right knee was completely flexed. The patella was pulled downwards and pushed upwards by placing both the thumbs over the inferior pole of the patella. Once the patella was relocated, the full range of movements of the right knee was normal without any patellar instability with intact patellar tendon. The reduction was cross-checked in the image intensifier, both in the anteroposterior and lateral views (Figures 6-7).

Quadriceps tendon repair

**Figure 8:**
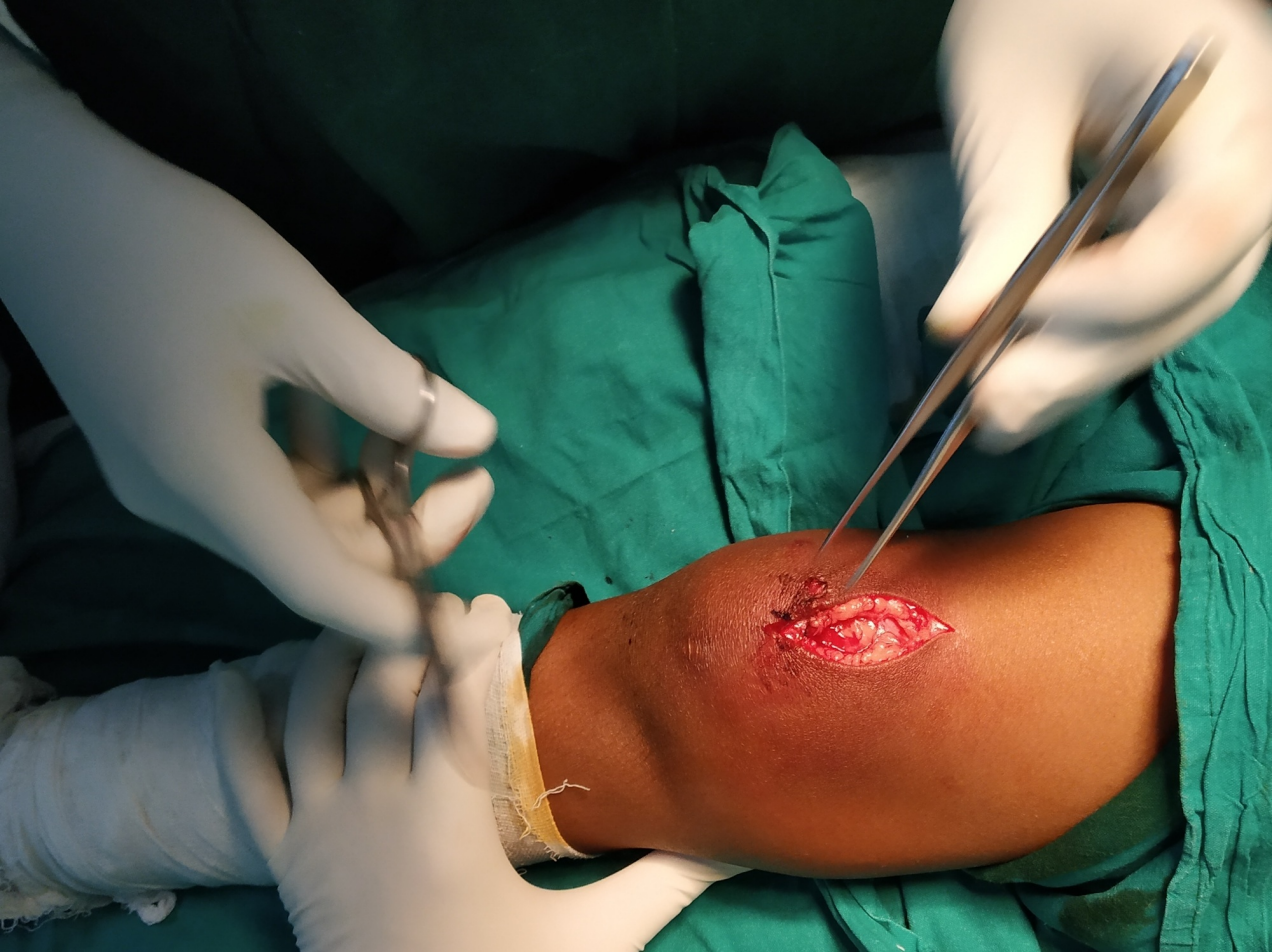
A paramedial incision given just above the superior pole of the right patella

**Figure 9:**
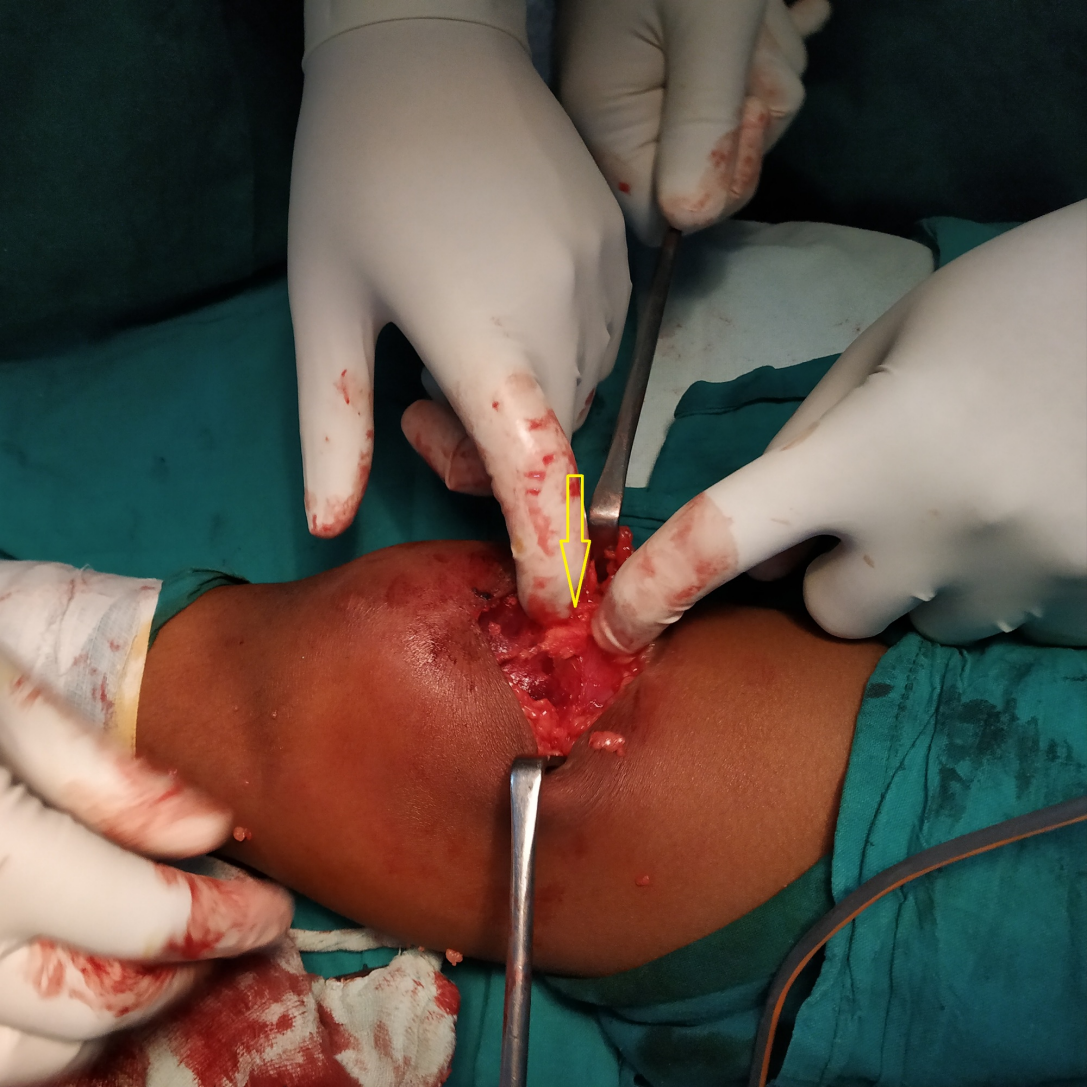
Quadriceps tendon tear identified in the superolateral aspect of the right patella

A para-medial incision was given longitudinally over the superior pole of the patella, extending proximally along the quadriceps (Figure 8). Bruising of the quadriceps muscle was noted, with an existing 40% tear in the superolateral aspect of the quadriceps tendon (Figure 9).

**Figure 10:**
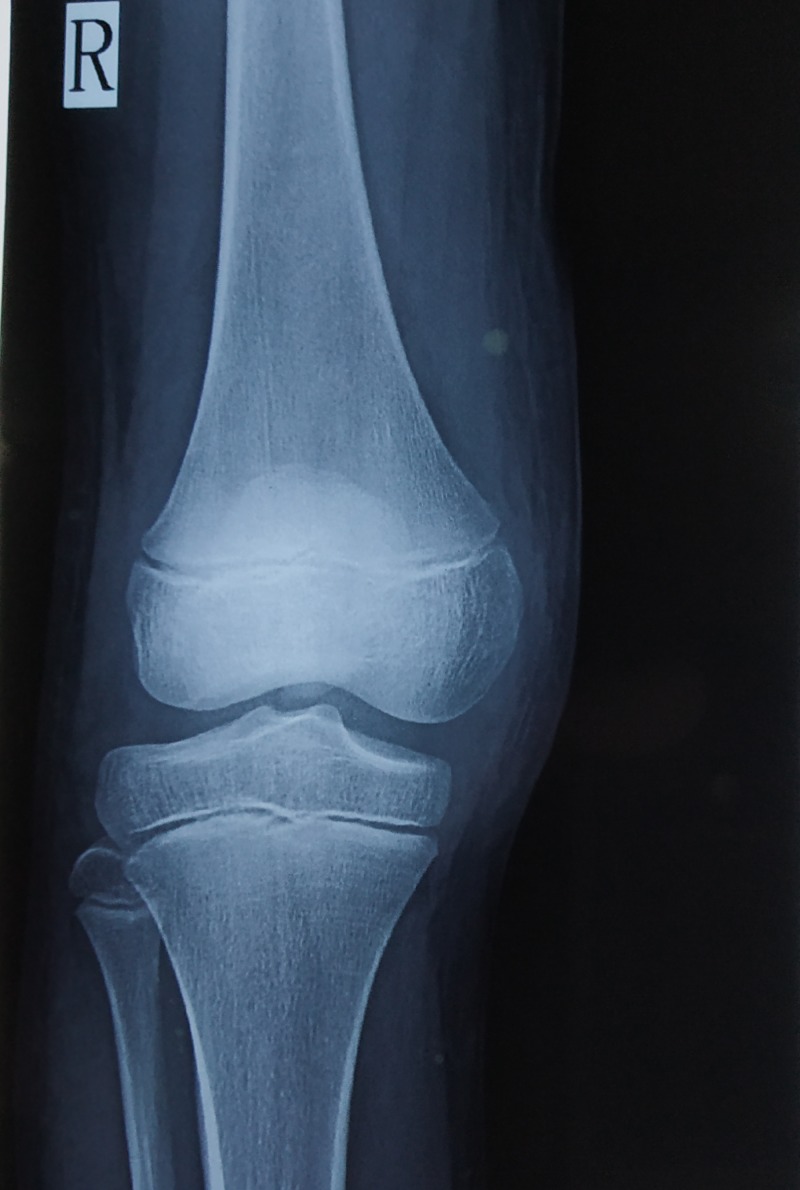
Post-operative X-ray of the right knee joint: anteroposterior view

**Figure 11:**
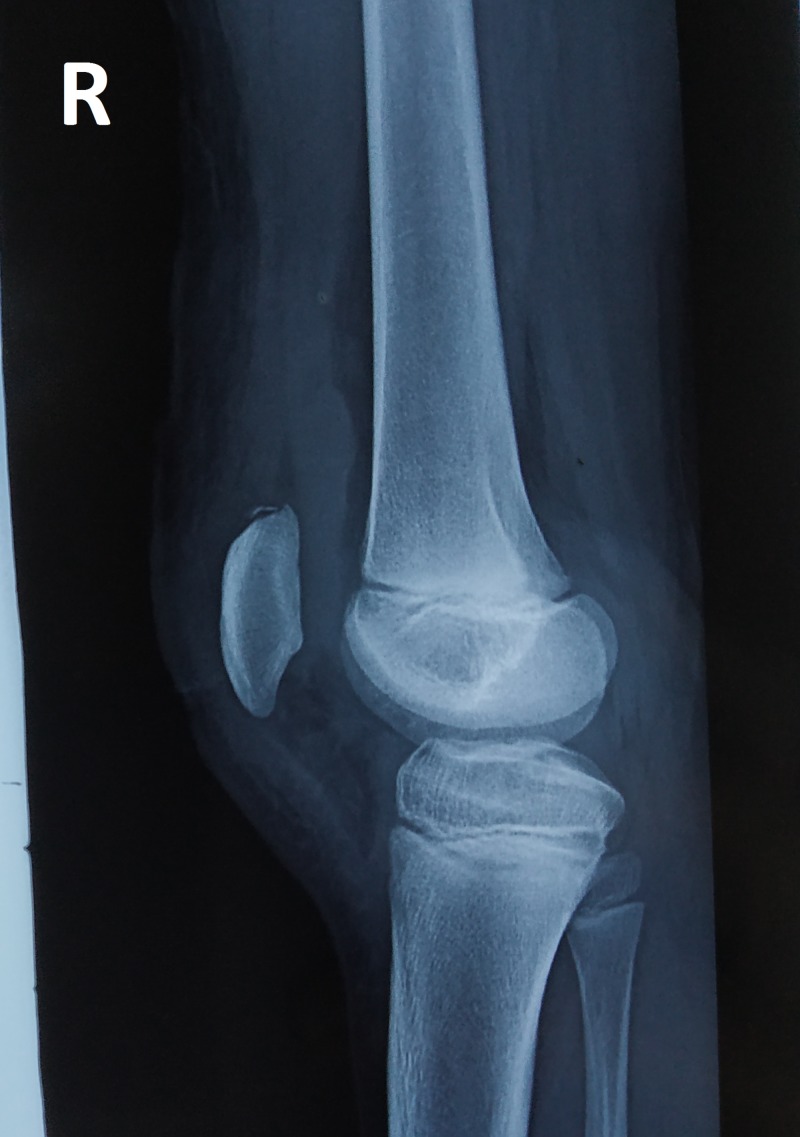
Post-operative X-ray of the right knee joint: lateral view

A non-absorbable polyethylene terephthalate suture (Ethibond, Ethicon Inc., New Jersey, United States) was used to repair the tendon tear. An above-knee cylindrical slab was applied in the complete extension of the right knee joint. The postoperative period was uneventful. The patient was allowed partial weight bearing on immediate post-op Day 1. Check X-rays of the right knee in the anteroposterior (AP) and lateral views were taken (Figures 10-11). Skin sutures were removed on the tenth postoperative day; the cylindrical slab was continued for three weeks. Follow-up was done in the first, second, and third months. Active quadriceps exercises of the right knee were started from the first follow-up. On every visit, the range of movements was satisfactory, with no visible deformity.

## Discussion

Patella is a sesamoid bone embedded in the quadriceps tendon; it rests in the patellofemoral groove. A direct blow to the superior pole of the patella with the knee in flexion is the most common cause of intra-articular dislocation. Numerous non-operative and operative methods have been described to treat a patella dislocation. 

Frangakis reported a case of an adolescent boy. In the study, it is explained that with the knee in flexion after a direct blow over the superior pole of the patella, there might be extreme movements of the patella into the tibiofemoral space of the knee joint. Subsequently, without leading to a tear in the tendon, a detachment of the tendon from the upper aspect of the patella occurs due to a reflex contraction of the quadriceps [3].

Berengera et al. opined that in order to avoid an iatrogenic quadriceps tendon tear and cartilage damage during closed reduction methods, surgical intervention would be desirable in case of complete rotation of the patella, thus allowing earlier knee mobilization and rehabilitation [4].

Madhavan et al., in their study of a young female with an associated injury to the ipsilateral femur from a co-existing patella dislocation, insisted on open reduction under general anesthesia, instead of a closed reduction under sedation [5]. Corso et al. reported a vertical type dislocation, where the patella got wedged laterally to the lateral femoral condyle. The patella rotated on its vertical axis of about 90 degrees. The occurrence of this type is five times less than that of horizontal type [6].

McHugh et al., in their study, reported that a reduction of the patella by closed manipulation under anesthesia resulted in a good prognosis with a view to obtain the mobility of the pre-injury status. Avulsion of deep fibers of the quadriceps tendon is caused by a direct blow onto the proximal pole of the patella. This might force the patella to rotate about a horizontal axis with the knee in flexion, thus causing the patella to hinge on the intact superficial fibers [2]. Pagdal et al., in their study, opined that in young patients with intra-articular patella dislocation but without any quadriceps or patellar tendon injury, recovery is good with conservative treatment or closed reduction [7]. While in our case, there was a co-existing quadriceps tendon tear.

A case report by Chauhan et al. explained that an intra-articular dislocation of the patella commonly occurs in adolescents and more commonly needs open reduction. There are two main methods of closed relocation of the patella. The first one involves upwards pressure over the patella and manipulation; the other method involves the initial hyperextension of the knee joint followed by passive flexion and manipulation. Due to the orientation of the patella, hyperextension is not achievable. Only in the locked position is the reduction maneuver on manipulating the patella possible [8].

The absolute management of acute patella dislocation following trauma in the young population is still in debate. All patients who present with acute patella dislocation are recommended to be investigated with MRI of the knee, to know the extent of the quadriceps tendon injury and the surrounding soft tissue status [9].

## Conclusions

Our case report aims to highlight that although intra-articular intercondylar patella dislocations are interpreted in literature, they are uncommon. We managed such a case of an adolescent by the closed maneuver and surgical treatment. The purpose of the surgical intervention was to assess and repair the co-existing quadriceps tendon tear, evidenced by ultrasound and MRI. Subsequently, the repair of the quadriceps tendon was performed, as a significant tear was noted. We aimed at earlier knee mobilization, rehabilitation, and progression towards the pre-injury status of the knee joint.
